# The Diagnostic and Prognostic Value of the Triglyceride-Glucose Index in Metabolic Dysfunction-Associated Fatty Liver Disease (MAFLD): A Systematic Review and Meta-Analysis

**DOI:** 10.3390/nu14234969

**Published:** 2022-11-23

**Authors:** Jing Wang, Shoumeng Yan, Yani Cui, Feinan Chen, Meihua Piao, Weiwei Cui

**Affiliations:** 1 Department of Anesthesiology, the First Hospital of Jilin University, Changchun 130021, China; 2 Department of Nutrition and Food Hygiene, School of Public Health, Jilin University, Changchun 130021, China; 3 School of Nursing, Jilin University, Changchun 130021, China

**Keywords:** diagnostic efficiency, threshold effect, cut-off, Meta-DISc, NAFLD/MAFLD

## Abstract

Metabolic dysfunction-associated fatty liver disease (MAFLD) has been related to a series of harmful health consequences. The triglyceride-glucose index (TyG index) appears to be associated with MAFLD. However, no consistent conclusions about the TyG index and incident MAFLD have been reached. PubMed, MEDLINE, Web of Science, EMBASE and the Cochrane Library were searched. Sensitivities, specificities and the area under the receiver operating characteristic (AUC) with a random-effects model were used to assess the diagnostic performance of the TyG index in NAFLD/MAFLD participants. Potential threshold effects and publication bias were evaluated by Spearman’s correlation and Deeks’ asymmetry test, respectively. A total of 20 studies with 165725 MAFLD participants were included. The summary receiver operator characteristic (SROC) curve showed that the sensitivity, specificity and AUC were 0.73 (0.69–0.76), 0.67 (0.65, 0.70) and 0.75 (0.71–0.79), respectively. Threshold effects (*r* = 0.490, *p* < 0.05) were confirmed to exist. Subgroup analyses and meta-regression showed that some factors including country, number of samples, age and disease situation were the sources of heterogeneity (*p* < 0.05). Our meta-analysis suggests that the TyG index can diagnose and predict MAFLD patients with good accuracy. The number of studies remains limited, and prospective studies are needed.

## 1. Introduction

With rapid economic growth, changes in lifestyle and dietary structure, and the prevalence of obesity, nonalcoholic fatty liver disease (NAFLD) has emerged as the most common liver disorder and is the most important cause of chronic liver diseases, involving a spectrum of liver diseases from simple steatosis to nonalcoholic steatohepatitis (NASH), liver fibrosis, cirrhosis and hepatocellular carcinoma [1]. The global incidence of NAFLD has progressively increased, and a meta-analysis of the incidence of NAFLD in 22 countries confirmed that its current global occurrence was 25.2%. Meanwhile, regional differences exist. The prevalence of NAFLD is 27.37% in Asia, 24.13% in North America, 31.79% in the Middle East (the region with the highest incidence) and 13.48% in Africa (the region with the lowest incidence) [2]. Younossi reported approximately 52 million individuals with NAFLD throughout Germany, Italy, France and the UK, incurring a total cost of EUR 35 B yearly, and the per-person direct medical charges for these nations have been estimated to vary from EUR 354 to 1163 [3]. NAFLD is associated with type 2 diabetes mellitus, hypertension, dyslipidaemia and cardiovascular disease [4], thus causing a severe clinical and economic burden worldwide. In addition, most patients suffering from NAFLD are asymptomatic, and early identification of NAFLD is vital for prevention and the establishment of public health policies. Notably, over the last two decades, as it is largely related to insulin resistance (IR) and associated disorders, a title change from NAFLD to metabolic dysfunction-associated fatty liver disease (MAFLD) has been proposed [5].

IR refers to decreased peripheral tissue insulin sensitivity, characterized by impaired glucose uptake and oxidation, and it participates in the key hyperlink of the pathogenesis of MAFLD [6,7]. MAFLD is viewed as the hepatic manifestation of metabolic syndrome, and as the foremost elements of metabolic syndrome, high triglycerides (TGs) and fasting plasma glucose (FPG) play key roles in the improvement of fatty liver [8,9]. The triglyceride glucose (TyG) index, a product of fasting blood glucose and triglycerides, has been recently proposed as a simple, low-cost surrogate marker of IR [10,11]. The results of some research have indicated that the TyG index could also be associated with MAFLD. Moreover, the TyG index can also be especially sensitive to the importance of steatosis due to the significance of increased triglycerides in liver fat accumulation [10]. However, whether the elevated TyG index indicates the presence of MAFLD remains controversial. Therefore, a meta-analysis was performed to consider the diagnostic and predictive value of the TyG index in MAFLD.

## 2. Materials and Methods

This systematic review and meta-analysis were performed in accordance with the “Preferred Reporting Items for Systematic Reviews and Meta-Analyses” (PRISMA) statement, which describes an evidence-based minimum set of items for reporting in systematic reviews and meta-analyses.

### 2.1. Sources and Methods of Data Retrieval

PubMed, MEDLINE, Web of Science, EMBASE and the Cochrane Library were searched from the inception of each database inceptions until 23 April 2022. The language or date of publication was not restricted. The terms ‘OR’ and ‘AND’ were used as the set operator to mix unique units of results. Additionally, potentially applicable research was screened from the reference lists of relevant research, systematic reviews or meta-analyses. The search strategy is shown in Table 1.

### 2.2. Inclusion Criteria and Exclusion Criteria

The inclusion criteria were as follows: (a) subjects: patients diagnosed with NAFLD/MAFLD (based on the corresponding clinical guidelines, which mainly include imaging methods such as ultrasound and laboratory biochemical indicators, but where alcohol as the cause was excluded), and all participants underwent TG and FPG detection; and (b) the diagnostic accuracy of the TyG index was reported, such as sensitivity, specificity, area under curve (AUC) and 95% confidence interval (CI). The exclusion criteria were as follows: (a) insufficient initial data to obtain true positives (TP), false positives (FP), true negatives (TN), false negatives (FN); (b) duplicate literature, in vitro studies and nonhuman studies; (c) reviews articles, editorials or letters; and (d) conference literature or case studies.

### 2.3. Data Extraction

Two researchers independently evaluated all relevant papers, extracted potentially eligible data, and discussed and resolved disagreements with relevant experts. The following data from all included relevant studies were extracted: (a) first author name, year of publication, country, study design, age, and number of participants; and (b) TP, FP, TN, FN, reference standards, cut-off values, AUCs and 95% CIs.

### 2.4. Quality Assessment

The methodological quality for the included literature used was evaluated independently using the Review Manager (Rev Man. Version 5.4. Copenhagen: The Nordic Cochrane Centre, The Cochrane Collaboration, 2014) with the aid of two investigators based completely on the Cochrane risk-of-bias criteria [12], which covered four domains (patient selection, index test, reference standard, flow and timing). Each domain was assessed in terms of risk of bias, and the first three domains were additionally assessed in terms of issues concerning applicability. Signaling questions were blanketed to assist in judging the risk of bias. The QUADAS-2 tool permitted a greater obvious ranking of bias and applicability of major diagnostic accuracy studies.

### 2.5. Statistical Analysis

Statistical analyses were conducted using the Meta-DISc version 1.4 and Stata (version 16.0, Stata Crop LLC, College Station, TX, USA). Heterogeneity was examined through Cochran’s Q-value and Higgins *I* squared (*I*^2^) [13,14]. *I*^2^ values of 25%, 50% and 75% indicate low, moderate and high heterogeneity, respectively [15]. *I*^2^ > 50% or *p* value < 0.05 indicated that heterogeneity existed, and a random-effects coefficient binary regression model was used [13]. Publication bias was visually assessed via Deeks’ funnel plot and asymmetry test, and it was regarded as existing if there was a nonzero slope coefficient (*p* < 0.10) [16].

In the meta-analysis of diagnostic test accuracy, one of the principal reasons for heterogeneity is the threshold effect, which arises from one-of-a-kind cut-off values or thresholds used in exceptional research to outline a positive (or negative) test result [14]. When a threshold effect exists, there is a negative correlation between sensitivities and specificities (or a positive correlation between sensitivities and 1-specificities) [17], which results in a typical pattern of a “shoulder arm” in a receiver operating characteristic (ROC) curve. Potential threshold effects have been investigated with the aid of Spearman’s correlation coefficient between the logit of sensitivity and logit of 1-specificity. A strong positive correlation would indicate a threshold effect, and a *p* value < 0.05 was considered statistically significant [14]. The heterogeneity of a study was due to the threshold effect; it was deemed appropriate to pool the accuracy records by fitting a summary receiver operating characteristic (SROC) curve [18]. A hierarchical summary receiver operating characteristic (HSROC) curve and bivariate boxplot were constructed with the summary points displayed.

The AUC was used to consider the capabilities of the TyG index to screen for MAFLD. An AUC of 0.75 to 0.92 is good. An AUC much less than 0.75 may additionally be reasonable; however, the test has evident shortcomings in its diagnostic accuracy [19].

The diagnostic odds ratio (DOR) test determines the ratio of the odds of positivity for efficaciously diagnosing a disease relative to the odds of positivity obtained in individuals with no sickness (false positivity). DOR is derived from logistic models, and it is feasible to consist of additional variables to correct for heterogeneity [20]. The Cochran Q-value of the DOR was used to test for non-threshold effects of variation. Using the strategies of restricted maximum likelihood estimation (REML) and inverse variance weighted least squares, we performed a subgroup analysis to find the source of heterogeneity. Design (retrospective or prospective), experiment (described experiment in detail or not clear), population (the population was described in detail versus approximately), country (China or other), number of samples (≥1000 or <1000), age (<18 or ≥18), baseline disease (yes or no), and reference standard (imaging examination or not) were used as the covariates. The factors observed to be strongly related to accuracy were then covered one by one in the bivariate model to examine the overall sensitivity and overall specificity between one-of-a-kind strata [15].

## 3. Results

### 3.1. Literature Search

**Figure 1:**
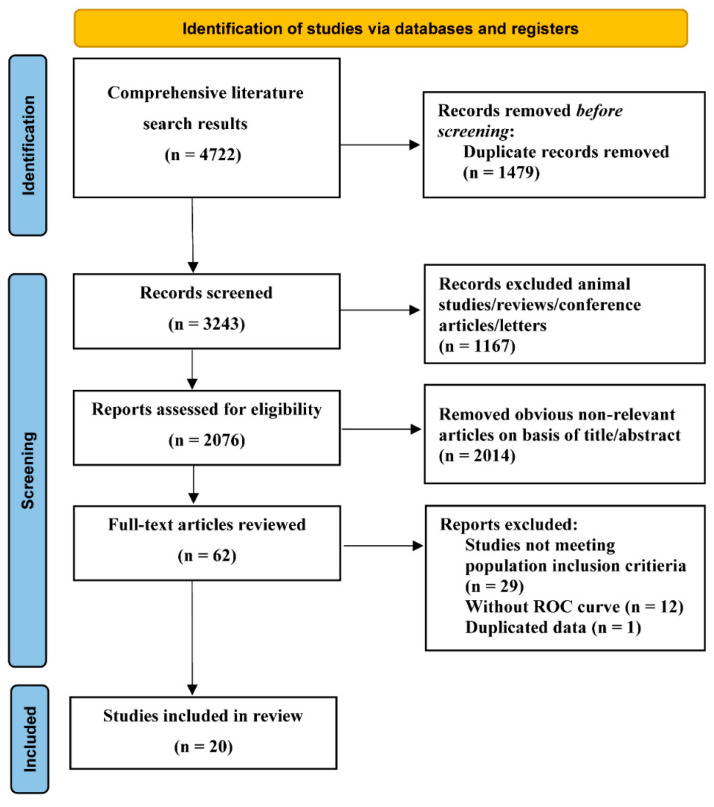
Flow graph of the literature search and selection.

The comprehensive computer literature search from PubMed, MEDLINE, Web of Science, EMBASE and the Cochrane Library revealed 4722 articles, as shown in Figure 1. Ultimately, 20 studies involving 165,725 participants were included [10,21,22,23,24,25,26,27,28,29,30,31,32,33,34,35,36,37,38,39].

### 3.2. Characteristics of the Included Studies

The detailed situation of the included studies is presented in Table 2. The regions included China [10,21,22,23,25,26,27,28,29,30,31,32,33,34] (n = 14), Iran [35,36] (n = 2), Korea [37,38,39] (n = 3) and Japan [24] (n = 1). Two studies [21,30] included only children, and 12 studies included only adults. Six studies had comorbid conditions (obesity [35,39], chronic kidney disease (CKD) [37], T2D [27], MetS [34], and obesity/T2D [36]). Two studies [29,31] were prospective studies. The other studies were retrospective studies.

**Figure 2:**
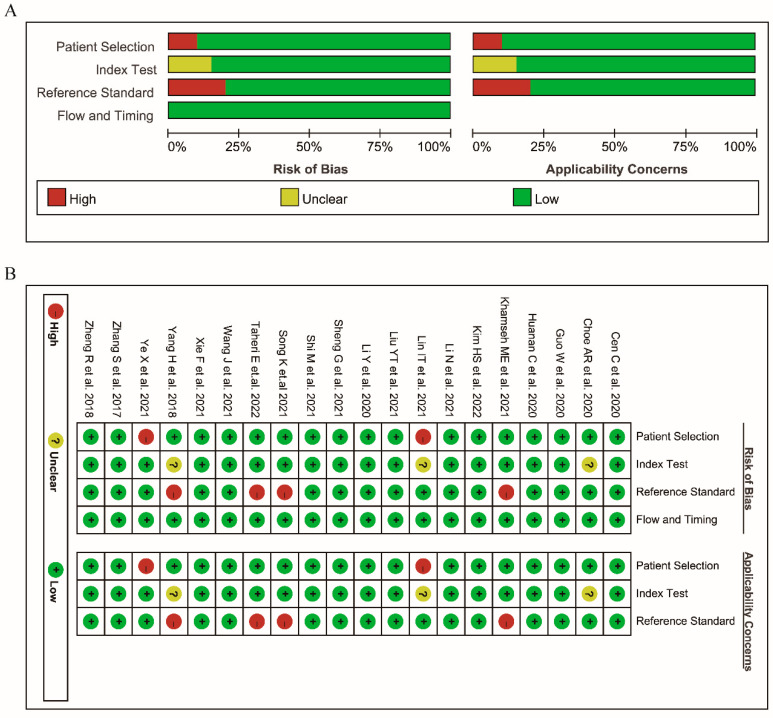
Methodological quality of included studies in accordance to the Quality Assessment of Diagnostic Accuracy Studies-2 (QUADAS-2) tool. (**A**) Overall assessment of the quality for included studies. (**B**) Methodological quality summary for included studies [10,21,22,23,24,25,26,27,28,29,30,31,32,33,34,35,36,37,38,39].

**Figure 3:**
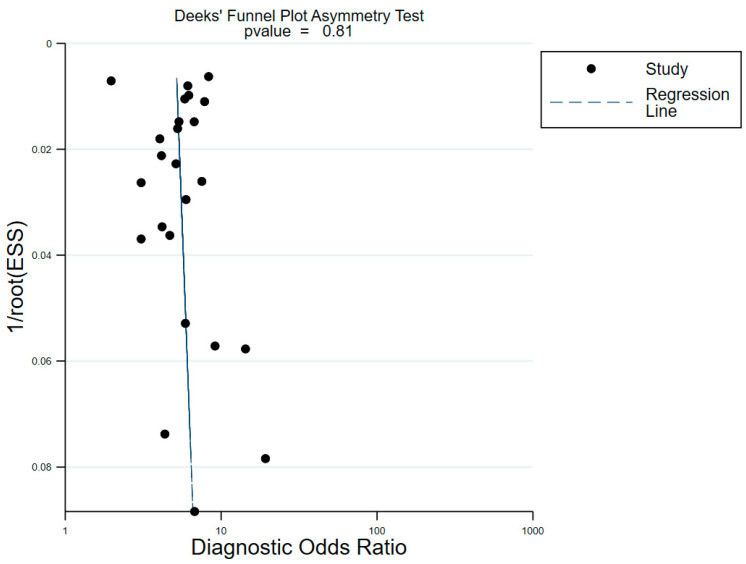
Deek’ funnel plot asymmertry test for assessing publication bias in the included studies with superimposed regression.

The risk of bias within the included studies was assessed via QUDAS-2 (Figure 2). According to QUDAS-2, all studies fulfilled more than 11 items from the 14-item QUDAS-2 checklist, which indicated overall high quality. Meanwhile, no significant publication bias was found (*p* = 0.81 for the slope coefficient), as shown in Figure 3.

### 3.3. Diagnostic Efficiency (Threshold Effect)

**Figure 4:**
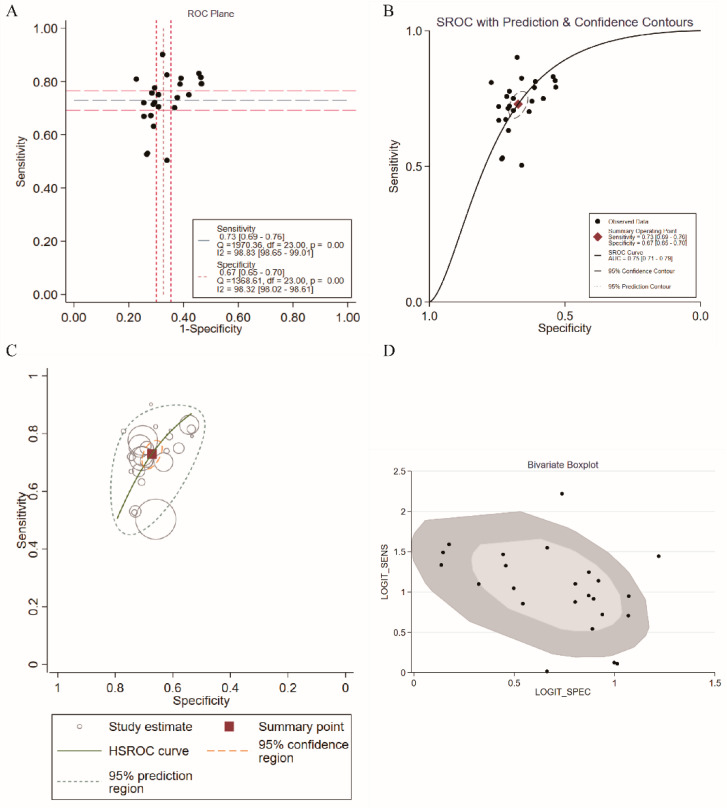
Pooled diagnostic parameters of TyG for MAFLD. (**A**) Representation of sensitivity against (1-specificity) in ROC curve for each study of TyG in the diagnosis and prediction for MAFLD. (**B**) SROC curve with pooled estimates of sensitivity and specificity and AUC of TyG in the diagnosis and prediction of MAFLD. (**C**) HSROC curve of diagnosis and prediction for MAFLD. (**D**) Bivariate Boxplot of the sensitivity and specificity in the include studies (The inner oval represents the median distribution, and the outer oval represents 95% CI boundary) (SROC: summary receiver operator characteristic; HSROC: hierarchical summary receiver operating characteristic; AUC: area under curve).

The results of our meta-analysis showed that the AUC of the ROC was 0.75. However, there was a positive correlation between sensitivities and 1-specificities in the ROC curve (*r* = 0.490, *p* = 0.015), resulting in a typical pattern of a “shoulder arm” plot, which indicated that a notable threshold effect existed (Figure 4A) and caused variations in accuracy estimates among the individual studies. Therefore, SROC and HSROC analyses were performed. In the SROC analysis, pooled sensitivity, and specificity with 95% CI were 0.73 (0.69–0.76) and 0.67 (0.65, 0.70), respectively (Figure 4B).

In addition, the HSROC curve (green line) is shown in Figure 4C, suggesting that the HSROC curve was asymmetrical (*β* = −0.44, 95% CI = −0.84, −0.05, *z* = −2.21, *p* = 0.027) and that the diagnostic and prognostic value of the TyG index in MAFLD was accurate (lambda = 1.70, 95% CI = 1.54, 1.85). Circles indicate study estimates, boxes denote summary points, blue dashed lines indicate 95% prediction regions, and orange dashed lines denote 95% confidence regions. The bivariate boxplot of Figure 4D shows the distribution of the results of all included studies. All of these results showed that the TyG index could diagnose and predict MAFLD with good diagnostic efficiency (AUC = 0.75, 95% CI = 0.71, 0.79). Meanwhile, some studies have suggested that the TyG index is a powerful tool for diagnosing and predicting the outcome of infected diseases when combined with other markers, such as body mass index (BMI) and waist circumference (WC). The specific details of the diagnostic and prognostic value of the TyG index and TyG index-related parameters are shown in Table 3.

### 3.4. Different Cut-Off Values of the TyG Index

**Figure 5:**
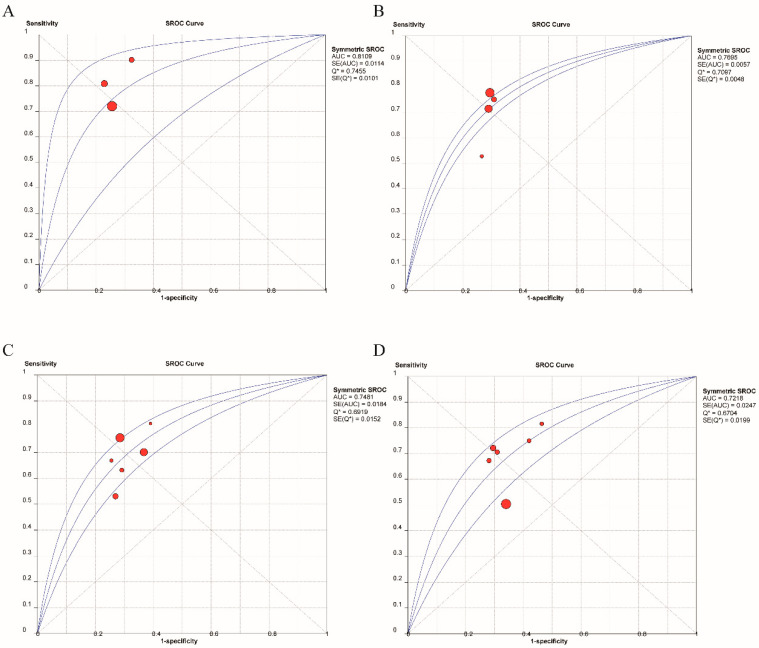
SROC curve with 95% confidence region and prediction region for the diagnosis of MAFLD. (**A**) cut off value with TyG < 6. (**B**) cut off value with 6 ≤ TyG < 8. (**C**) cut off value with 8 ≤ TyG < 8.5. (**D**) cut off value with TyG ≥ 8.5 (AUC: area under curve).

The presence of threshold effects could lead to differences in sensitivity and specificity. The cut-off values of this meta-analysis ranged from 0.146 to 8.7 (the data in four studies were missing). To assess the diagnostic performance of the TyG index at different cut-off values, subgroup analyses were conducted (cut-off values of TyG: <6 (n = 3), 6–8 (n = 4), 8–8.5 (n = 6), and ≥8.5 (n = 6)). The detailed results are shown in Table 4. For the TyG cut-off < 6 group, the pooled sensitivity and specificity with 95% CI were 0.75 (0.71, 0.78) and 0.74 (0.72, 0.76), respectively. The AUC was 0.81 ± 0.01, and a high diagnostic value was found. Meanwhile, we found that with the increase in the TyG cut-off value, the diagnostic value of TyG for MAFLD decreased. Therefore, the determination of the optimal cut-off value needs to be combined with more data and specific clinical situations (Figure 5, Table 4).

### 3.5. Non-Threshold Effect

**Figure 6:**
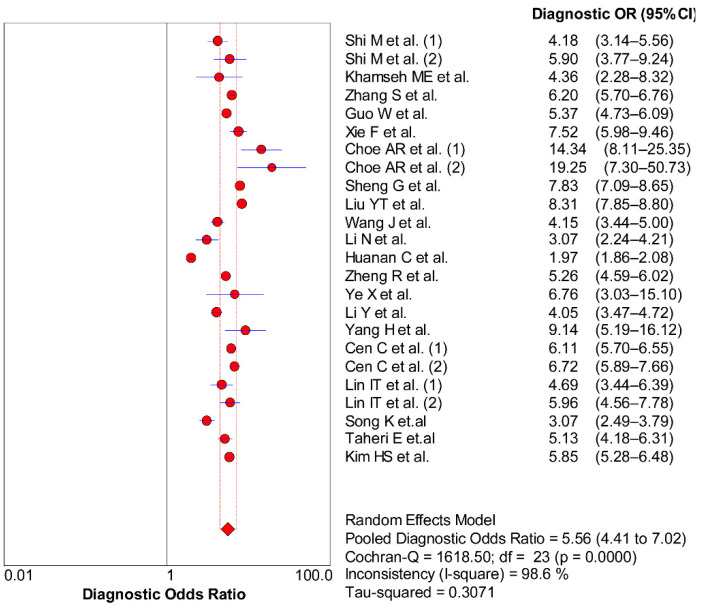
Forest plot of DOR with corresponding 95% CI of TyG for MAFLD [10,21,22,23,24,25,26,27,28,29,30,31,32,33,34,35,36,37,38,39].

**Figure 7:**
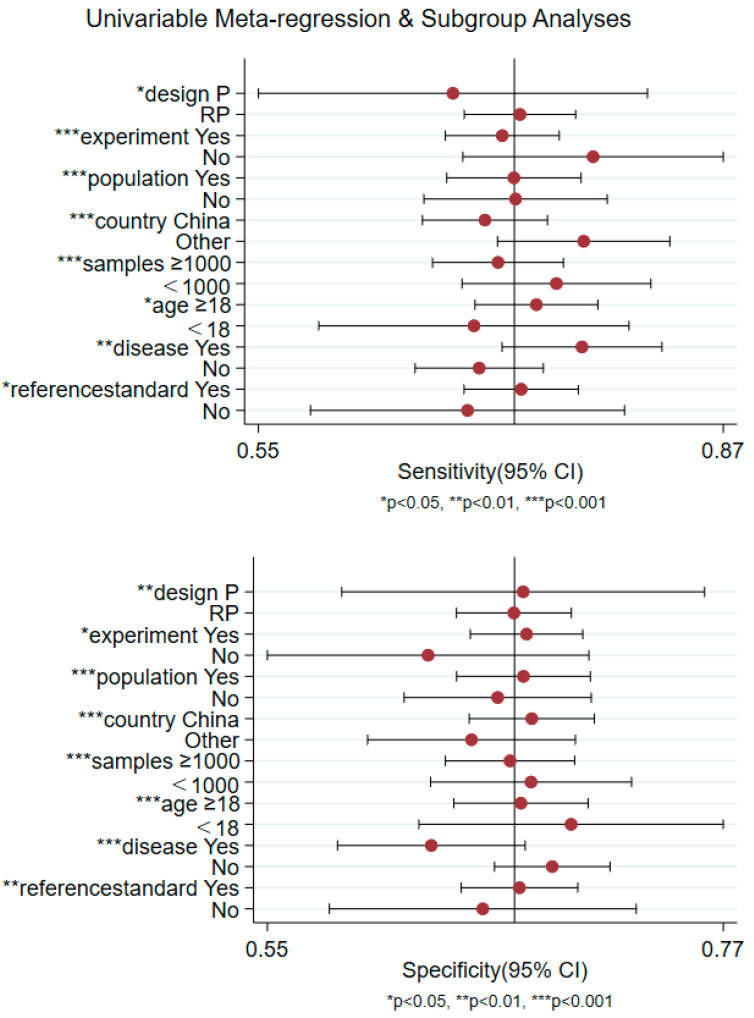
Univariable meta-regression and subgroup analyses for different variables to discover sources of heterogeneity (RP, retrospective; P, prospective).

As shown in Figure 6 (DOR = 5.56, 95% CI = 4.41, 7.02, *Q* = 1618.50, *p* < 0.001), part of the heterogeneity could also be due to non-threshold effects. We further found the source of heterogeneity through sensitivity analysis, subgroup analysis and meta-regression. The results indicated that including the country, number of samples, age and disease situation were the sources of sensitivity and specificity heterogeneity (*p* < 0.05) (Figure 7).

## 4. Discussion

Past studies have demonstrated that obesity, metabolic disorders, and environmental elements lead to the incidence and improvement of MAFLD. The prevalence of MAFLD is growing rapidly, bringing a host of adverse consequences [2,40,41]. A variety of indices have been found for the early detection of NAFLD, such as the fatty liver index, NashTest, hepatic steatosis index, SteatoTest, OxNASH score, aspartate aminotransferase/alanine aminotransferase (AST/ALT) ratio, enhanced liver fibrosis panel (ELF), aspartate aminotransferase to platelet ratio (APRI) and fibrosis-4 score (FIB-4) [42,43,44,45,46]. However, due to the complex calculation among variables and high cost, it is difficult for the above indices to be extensively used in clinical practice [47]. The mathematical model of the TyG index was first derived by Simental and other scholars for assessing the situation of IR [48,49,50]. As the calculation of TyG only requires triglyceride and fasting blood glucose, it is very suitable for large-scale epidemiological investigation [48]. Meanwhile, existing evidence suggests that triglycerides and fasting blood glucose are involved in the formation of fatty liver, and the theory of IR is considered to be important in the pathogenesis of MAFLD. Moreover, the TyG index is closely associated with NAFLD, and the TyG index is regarded as an effective, practical, and low-cost device to identify individuals at risk of hepatic steatosis with excessive sensitivity and specificity [47]. Therefore, the TyG index could be a good diagnostic index for MAFLD.

Existing studies have demonstrated the diagnostic value of some of the above indices in related liver diseases. A study revealed that the AUCs of the FIB-4 index, NAFLD fibrosis score (NFS) and BARD score for predicting advanced fibrosis in NAFLD were 0.744, 0.702 and 0.733, respectively [51]. In addition, a meta-analysis proposed that ARPI and FIB-4 should detect hepatitis B-related fibrosis with moderate sensitivity and accuracy [52]. However, given the limited sample size and power, we performed a meta-analysis to estimate the diagnostic value of the TyG index effectively. The results indicated that the TyG index had sensitivity (73%) and specificity (67%) in diagnosing MAFLD, with a pooled AUC of 0.75. Meanwhile, TyG index-related parameters are the combined parameters of the TyG index with BMI, WC, and waist-height ratio (WHtR), which were first reported by Ko et al. [53]. They proposed that TyG index-related parameters had the highest AUC value for predicting IR in contrast to visceral weight problems, lipid parameters and adipokines. Among them, TyG-BMI has the largest AUC in identifying IR, and it has also been found to be strongly associated with cardiovascular and cerebrovascular diseases such as hypertension and ischaemic stroke [54,55]. Additionally, compared with the TyG index and (homeostasis model assessment) HOMA-IR, TyG index-related parameters, including TyG-BMI and TyG-WC, showed better detection ability for MAFLD, liver fibrosis and moderate-to-advanced fibrosis, especially in younger people and diabetes patients [56]. Notably, TyG-BMI shows excellent predictive performance in detecting NAFLD in young and middle-aged people, and the correlation between liver fibrosis and TyG-BMI was stronger [35,57]. Importantly, as TyG-BMI is calculated based on FPG, TG and BMI, it is easy to obtain in the clinic, which is conducive to rapid promotion and application [57]. Our meta-analysis was consistent with previous results. However, due to the limited number of articles, more research focusing on TyG-related parameters is needed to assess its value.

Owing to the terrific threshold effect, fitting the SROC and HSROC curves was considered to be a more suitable method of evaluating the diagnostic value. Meanwhile, although the threshold effects could lead to the source of heterogeneity in our study, nonthreshold effects were also found. Meta-regression and subgroup analyses were performed to assess the potential impact factors, including population, disease, country, samples and age. Our results confirmed that these factors contributed to the existence of heterogeneity. In addition, the range of optimal cut-off values for the included studies was wide, possibly due to differences in sample size and age among the included studies. Sheng et al. proposed that the TyG index had higher predictive overall performance in the younger populace (age 18–30 years) [24].

Some inherent limitations existed in our study design and need to be viewed when deciphering our results. First, although we also found some studies from Mexico [47,58], Japan [59], and Brazil [60] that assessed the relationship between the TyG index and NAFLD, these studies were excluded due to the lack of necessary diagnostic data. All studies covered in our meta-analysis were from Asia, which may have potentially biased the results. Meanwhile, most of the studies included in this meta-analysis were retrospective observational studies, and the sample size of these studies varied widely. In addition, significant heterogeneity was discovered, and the threshold effect existed in our meta-analysis.

## 5. Conclusions

All currently available evidence from our meta-analysis suggests that the TyG index can diagnose and predict MAFLD patients with good accuracy. However, the number of studies remains limited, and prospective studies are needed to find a specific cut-off value of diagnosis.

## Figures and Tables

**Table 1:** Search strategy.

Criteria	Descriptions and Search Terms Used for each Criteria
NAFLD/MAFLD	Non-alcoholic fatty liver OR Non alcoholic Fatty Liver Disease OR Nonalcoholic Fatty Livers OR NAFLD OR Nonalcoholic Fatty Liver Disease OR Nonalcoholic OR Nonalcoholic Steatohepatitis OR nonalcohol-related fatty liver disease OR non-alcohol-related fatty liver disease OR non-alcohol related fatty liver disease OR Nonalcoholic Steatohepatitides OR NASH OR nonalcoholic fatty liver disease OR Nonalcoholic Fatty Liver Disease OR Nonalcoholic Steatohepatitis OR non-alcoholic steatohepatitis OR fatty liver OR NASH/non-alcoholic steatohepatitis OR nonalcohol-related fatty liver disease OR non-alcohol related fatty liver disease OR Metabolic dysfunction-associated fatty liver disease OR MAFLD OR MAFLD-related cirrhosis OR metabolic associated fatty liver disease
TyG index	triglyceride-glucose index OR triglyceride glucose index OR TyG index OR triglyceride and glucose index OR triglyceride–glucose (T/Gly) index OR TyGs OR triglyceride glucose indices OR The triglyceride-glucose index OR Triglyceride/glucose index OR Triglycerides and glucose index OR triglycerides/glucose Index (TyG Index)

**Table 2:** Characteristics of Studies Included in the meta-analysis of TyG index in NAFLD/MALFD participants.

Author	Year	Country	Age	Disease	AUC (95% CI)	Design	Reference Standard	Samples	Cut-Off	tp	fp	fn	tn
Mengte Shi et al [21]. (1)	2021	China	≤18	no	0.733 (0.698, 0.768)	RP	ultrasonography	1291	8.42	165	300	96	730
Mengte Shi et al [21]. (2)	2021	China	≤18	no	0.769 (0.719, 0.818)	RP	ultrasonography	554	8.42	75	113	37	329
Mohammad E. Khamseh et al [35].	2021	Iran	30–65	obesity	0.676 (0.598, 0.754)	RP	Transient elastography	184	-	76	41	20	47
Shujun Zhang et al [10].	2017	China	≥20	no	0.782 (0.773, 0.790)	RP	ultrasonography	10,761	8.5	3140	1892	1209	4520
Wen Guo et al [22].	2020	China	≥18	no	0.761 (0.747, 0.774)	RP	ultrasonography	4784	8.7	2049	582	853	1300
Fangfei Xie et al [23].	2021	China	≥18	no	0.807 (0.785, 0.828)	RP	ultrasonography	1748	4.75	379	312	147	910
A Reum Choe et al [37]. (1)	2020	Korea	≥18	CKD	0.85 (0.80, 0.90)	RP	ultrasonography	567	0.146	72	109	17	369
A Reum Choe et al [37]. (2)	2020	Korea	≥18	CKD	0.84 (0.78, 0.90)	RP	ultrasonography	252	0.146	46	65	5	136
Guotai Sheng et al [24].	2021	Japan	-	no	0.8084 (0.7996, 0.8173)	RP	ultrasonography	14,251	8.2059	1899	3348	608	8396
Liu Yiting et al [25].	2021	China	18–80	no	0.816 (0.811, 0.820)	RP	ultrasonography	25,535	6.9	9022	4104	2595	9814
Jingrui Wang et al [26].	2021	China	19–93	no	0.725 (0.705, 0.746)	RP	ultrasonography	3239	8.55	534	1061	178	1466
Nong Li et al [27].	2021	China	-	T2D	0.651 (0.611, 0.691)	RP	ultrasonography	826	6.5	291	73	261	201
Chen Huanan et al [28].	2020	China	>60	no	0.60 (0.58, 0.61)	RP	ultrasonography	46,693	8.63	2851	13,951	2809	27,082
Rongjiong Zheng et al [29].	2018	China	≥18	no	0.76 (0.74, 0.77)	P	ultrasonography	4539	8.52	935	885	455	2264
Xiaolin Ye et al [30].	2021	China	3–14	no	0.765 (0.682, 0.835)	RP	ultrasonography	134	8.16	52	25	12	39
Yaling Li et al [31].	2020	China	14–90	no	0.7264 (0.7096, 0.7433)	P	ultrasonography	9767	8.3219	590	3280	251	5646
Huanhuan Yang et al [32].	2018	China	>60	no	0.793	RP	ultrasonography	918	-	80	123	17	239
Chao Cen et al [33]. (1)	2020	China	18–75	no	0.774 (0.767, 0.781)	RP	ultrasonography	16,468	6.95	4470	2960	1791	7247
Chao Cen et al [33]. (2)	2020	China	18–75	no	0.783 (0.769, 0.796)	RP	ultrasonography	5000	6.87	1320	1002	439	2239
I-Ting Lin et al [34]. (1)	2021	China	-	MetS	0.697	RP	ultrasonography	764	-	262	155	92	255
I-Ting Lin et al [34]. (2)	2021	China	-	MetS	0.747	RP	ultrasonography	1205	-	373	284	99	449
Kyungchul Song, et.al [38].	2021	Korea	10–19	no	0.667 (0.636, 0.697)	RP	ALT (>26 U/L for boys and >22 U/L for girls)	3728	8.391	215	895	190	2428
Ehsaneh Taheri et.al [36].	2022	Iran	35–70	obesity/T2D	0.862 (0.856, 0.877)	RP	Fatty liver index ≥ 60 Overweight or obese/T2DM	1932	8.62	790	447	178	517
Hwi Seung Kim et al [39].	2022	Korea	-	obesity	0.770 (0.762, 0.778)	RP	ultrasonography	10,585	-	2728	3331	556	3970

RP, retrospective; P, prospective; fn, False Negative; fp, False Positive; tn, True Negative; tp, True Positivity.

**Table 3:** Summary diagnostic accuracy at Various Diagnostic Parameters for Diagnosis and Prediction for MAFLD.

	Sensitivity	Specificity	AUC
TyG-BMI	0.79 (0.73, 0.84)	0.75 (0.63, 0.84)	0.84 (0.80, 0.87)
TyG-WC	0.82 (0.74, 0.88)	0.69 (0.61, 0.76)	0.81 (0.77, 0.84)
TyG	0.73 (0.69, 0.76)	0.67 (0.65, 0.70)	0.75 (0.71, 0.79)

**Table 4:** Summary diagnostic accuracy at Various Diagnostic Thresholds for Diagnosis and Prediction for MAFLD.

	Cut-Off < 6 (n = 3)	Cut-Off 6–8 (n = 4)	Cut-Off 8–8.5 (n = 6)	Cut-Off ≥ 8.5 (n = 6)
Pooled sensitivity (95%CI)	0.75 (0.71, 0.78)	0.75 (0.74, 0.75)	0.72 (0.70, 0.73)	0.64 (0.64, 0.65)
Pooled specificity (95%CI)	0.74 (0.72, 0.76)	0.71 (0.70, 0.71)	0.69 (0.68, 0.69)	0.66 (0.66, 0.67)
Pooled PLR (95%CI)	3.01 (2.60, 3.48)	2.47 (2.33, 2.63)	2.22 (1.87, 2.63)	1.99 (1.61, 2.45)
Pooled NLR (95%CI)	0.27 (0.17, 0.44)	0.41 (0.33, 0.51)	0.45 (0.35, 0.59)	0.45 (0.33, 0.62)
DOR (95%CI)	11.39 (6.30, 20.57)	5.98 (4.69, 7.63)	4.92 (3.33, 7.26)	4.40 (2.63, 7.37)
DOR (Cochran Q-value), (P)	7.09 (0.029)	75.03 (<0.001)	96.32 (<0.001)	661.86 (<0.001)
Spearman (P)	0.50 (0.667)	0.80 (0.200)	0.60 (0.208)	0.60 (0.208)
AUC (SE)	0.81 ± 0.01	0.77 ± 0.01	0.75 ± 0.02	0.72 ± 0.02

PLR: Positive Likelihood Ratio; NLR: Negative Likelihood Ratio.

## Data Availability

Not applicable.
